# Supplementary figures and images for: Antioxidant Activity and Seasonal Variations in the Composition of Insoluble Fiber from the Cladodes of Opuntia ficus-indica (L.) Miller: Development of New Extraction Procedures to Improve Fiber Yield
Source: Plants (Basel). 2024 Feb 16;13(4):544. doi: 10.3390/plants13040544 (PMC10892137; doi:10.3390/plants13040544)

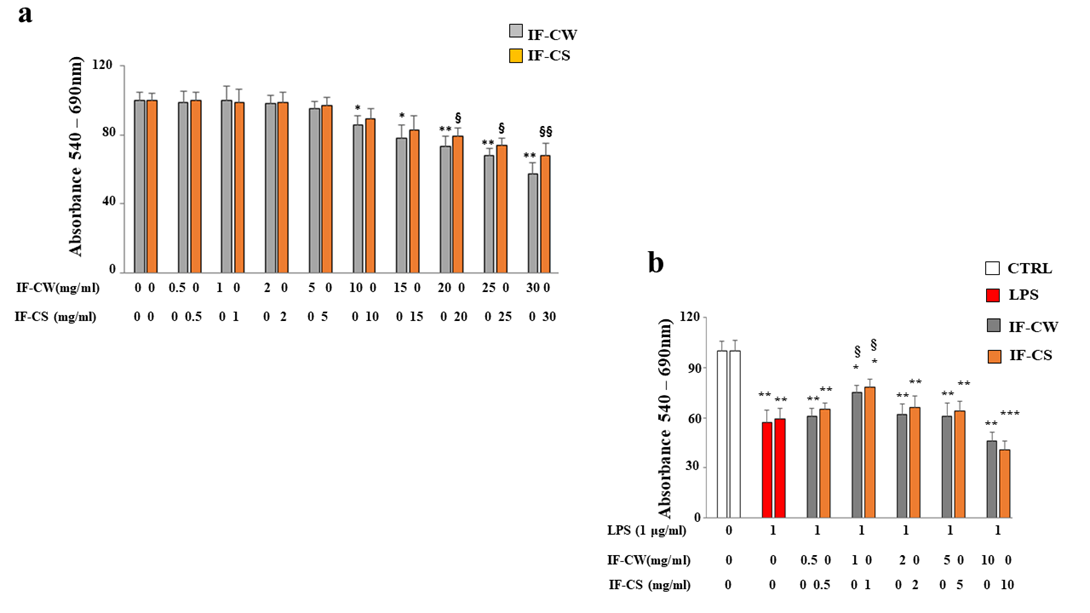

Supplement: Supplementary file 1 [file plants-13-00544-s001.zip › plants-2830424-supplementary.tif]
